# The Effect of Functional Electrical Stimulation and Therapeutic Exercises on Trunk Muscle Tone and Dynamic Sitting Balance in Persons with Chronic Spinal Cord Injury: A Crossover Trial

**DOI:** 10.3390/medicina55100619

**Published:** 2019-09-21

**Authors:** Margot Bergmann, Anna Zahharova, Märt Reinvee, Toomas Asser, Helena Gapeyeva, Doris Vahtrik

**Affiliations:** 1 Institute of Sport Sciences and Physiotherapy, University of Tartu, 51008 Tartu, Estonia; 2 Institute of Technology, Estonian University of Life Sciences, 51006 Tartu, Estonia; 3 Institute of Clinical Medicine, University of Tartu, 50406 Tartu, Estonia

**Keywords:** spinal cord injury, functional electrical stimulation, therapeutic exercise, muscle tone, dynamic sitting balance

## Abstract

*Background and objectives:* Functional electrical stimulation (FES) has shown good results in improving static and dynamic sitting balance in persons with spinal cord injuries. There is limited information about how regular surface FES combined with therapeutic exercise (TE) affect dynamic sitting balance and muscle tone. The objective of this study was to evaluate the effectiveness of a six-week physical therapy program consisting of FES and TE on muscle tone and sitting balance in persons with spinal cord injury (SCI). It was also important to explore the relationship between muscle tone and dynamic sitting balance. The third objective was to assess the change of characteristics over a six month period, when no intervention was carried out. *Material and methods:* Five men with SCI were alternately allocated to two study groups: SCI_FES+TE and SCI_TE. Eight healthy control group participants were recruited to collect reference data. SCI participants’ intervention lasted for six weeks in their homes. SCI_FES+TE conducted exercises with FES applied on erector spinae (ES) and rectus abdominis (RA) muscles. SCI_TE conducted exercises only. Muscle oscillation frequency (MOF; characterizing muscle tone) and limits of stability (LOS; characterizing sitting balance) were measured. A crossover study design was used. The time between the initial intervention and the crossover was seven months (ClinicalTrials registration ID NCT03517787). *Results:* MOF in SCI_FES+TE increased by 6.0% for ES and 6.1% for RA muscles. LOS of flexion increased 30.1% in SCI_FES+TE. Increase in lateral directions was similar for both study groups. Moderate to high negative correlation was found between MOF and LOS. After seven months, MOF of ES decreased 0.8%, MOF or RA decreased 1.4%, LOS of flexion decreased 31.9%, and LOS of lateral flexion to the left decreased 46.4%. *Conclusions:* The six-week therapy program combining FES and TE increased trunk muscle tone and dynamic sitting balance in flexion more than TE alone. Higher antagonist muscle tone negatively affects dynamic sitting balance and center of pressure (COP) trajectory distance in various directions. After seven months, a slight decline in trunk muscles tone values and an extensive decrease in sitting balance values were noticed.

## 1. Introduction

Traumatic spinal cord injury (SCI) may lead to long-term disability, which can have an impact on a person’s psychological, social, and physical well-being, and therefore, also on quality of life [1,2]. Among other factors, an SCI sufferer’s quality of life depends on the person’s dynamic sitting balance and the ability to sit unsupported [3,4], together with upper extremity function [5]. Due to the changes in sensation, muscle paralysis, and presence of spasticity, persons with SCI can have severely impaired postural control and balance [6,7]. As tonic activation of trunk muscles is required to maintain an upright sitting position and phasic activation is required to maintain the position while resisting balance disturbances [8], paralysis of trunk muscles is one of the most important factors affecting sitting balance [4]. Trunk, hip, and lower extremity muscle synergies are responsible for postural stability, thus, the paralysis of these muscles causes impairment of sitting balance [9]. Stretch reflexes, intermuscular reflexes, and intrinsic muscle properties are also responsible for trunk stability [10].

A muscle’s structure is composed of connective tissue and muscle fibers—both affect muscle tone [11]. The tone itself results from neural and non-neural (intrinsic) aspects [12]. Muscle oscillation frequency (MOF; Hz) describes the intrinsic tension of the muscle in the resting or passive state. Muscle tone reflects the possibility to restore the muscle’s physiological properties between contractions, and is measured without any voluntary contraction [11,13]. Muscle tone is altered in neurological conditions [12], including persons with SCI. It is difficult to assess muscle tone in clinical settings, as different scales (e.g., Modified Ashworth scale and Tardieu scale) are subjective and laboratory techniques (e.g., ultrasound imaging and magnetic resonance elastography) are not clinically feasible [12,13]. Muscle tone can be assessed using myotonometry [11,12,13]. The MyotonPRO device has shown good reliability in assessing person with SCI, therefore, it can be used to measure muscle properties over time [13].

Physical therapy programs for persons with SCI typically incorporate therapeutic exercises (TE), along with practicing functional activities in a seated position. Therapy follows the principles of motor re-learning [6,14]. Strength training is commonly used to improve muscle activation, but in the case of SCI, functional electrical stimulation (FES) can also be applied to improve muscle recruitment [15]. FES is used to elicit muscle contractions in upper motor neuron lesions, and thus, provide increases in trunk stability [3,15].

Despite the importance of persons with SCI having seated postural stability, only a few biomechanical studies have analyzed quantitative data [9]. Force plates are mainly used to register the center of pressure (COP) displacement [16], however, force-sensing surfaces underneath the left and right buttock have also been used [9]. There is limited information examining links between intrinsic muscle tension and sitting balance, and on whether a physical therapy program of concurrent FES and TE influences both characteristics. The objective of the study was to evaluate the effectiveness of a six-week physical therapy program consisting of FES and TE on muscle tone and sitting balance in persons with SCI. It was also important to explore the relationship between muscle tone and dynamic sitting balance. The third objective was to assess the changing of characteristics over a seven-month period, when no intervention was carried out.

## 2. Materials and Methods

This crossover trial was conducted at the University of Tartu between March 2017 and October 2018. The study protocol is registered in the United States National Library of Medicine (ClinicalTrials.gov), ID NCT03517787, protocol ID Spinaal. All applicable institutional and governmental regulations concerning the ethical use of human volunteers were followed during this research. The current study was approved by Research Ethics Committee of the University of Tartu (permit number: 263/T-4, 17.10.2016). All patients signed a written informed consent for participation in the study.

### 2.1. Participants

Data from 18 participants were transmitted from the university hospital to the main author of the study. The suitability for the study was initially specified with a telephone interview, followed by consultation with a neurosurgeon who confirmed that the participant can be included in the study. Inclusion criteria for the study participants were: (1) age 18–55 years; (2) chronic SCI (≥12 months’ post injury) [17]; (3) traumatic origin of the injury located in the cervical region, resulting in tetraplegia; (4) scoring of B or C by American Spinal Injury Association Impairment Scale (AIS); (5) able to sit unsupported on a custom-made chair. Participants were excluded from the study if they had cardiopulmonary insufficiency, cardiac pacemaker, oncological disease, epilepsy, open wounds, or metal implants in the area where FES was planned to be performed. Eleven participants were excluded from the study due to inadequacy of inclusion criteria. Seven were alternately allocated to SCI_FES+TE and SCI_TE study groups, but due to health complications during the first six-week intervention, two more participants had to be excluded from the study. A total of five SCI participants completed the study. Participants were alternately assigned to two groups: group 1 performed simultaneous TE and FES on erector spinae (ES) and rectus abdominis (RA) muscles, while group 2 performed only TE. As the crossover study design was used, randomization was used for only the first intervention. After seven months, participants enrolled in the opposite intervention group, ensuring that both groups had equal sample sizes. In the current study, the groups were marked accordingly: SCI_FES+TE, SCI_TE, and control group (CG). Due to the small sample size and crossover study design, participants were the same in both SCI study groups. Participants who were initially assigned to FES+TE group were assigned seven months later to TE group, and vice versa. Anthropometric data was measured only once as a baseline, and the same data were used seven months later.

Eight control group (CG) participants were recruited from the employees of the University of Tartu, whose gender and age were matched with SCI participants’ respective characteristics. Inclusion criteria for CG participants were a sedentary lifestyle and that they were not supposed to be physically active during leisure time. Exclusion criteria were the same for CG as they were for the SCI participants. Participants’ demographic data, duration, and classification of SCI are presented in Table 1.

### 2.2. Intervention

The therapy program consisted of eight different exercises aimed at improving sitting balance and upper body posture. The program lasted for 6 weeks and therapy was carried out twice a week. Twelve repetitions were performed for each exercise, with 3 sets performed for the first 3 weeks and 4 sets for the last three. SCI participants’ training and data collection were conducted in their homes. Exercises were conducted in their own wheelchair with the guidance of a physical therapist. CG carried out only one round of therapy to collect reference data. Training and data collection for CG took place on the university premises. SCI_TE performed exercises only, while SCI_FES+TE performed the same exercises, but additionally FES was delivered.

FES was used to induce muscle contraction in ES and RA. A preset program from a T-One Rehab (I-Tech Medical Division, Martellago, Italy) four-channel FES device was selected to deliver transcutaneous electrical stimulation using 5 × 5 cm self-adhesive gel electrodes (I-Tech Medical Division, Martellago, Italy). The preset program was chosen because the overall aim was that participants could continue the training using the same parameters. The program was selected mainly according to the frequency parameters. Lower frequency parameters postpone fatigue and discomfort [18], and they are also in compliance with previous studies [19]. Electrodes were placed on the thoraco-lumbar area of the ES and RA muscles bilaterally. ES and RA muscles were chosen for stimulation as these muscles have been shown to contribute significantly to trunk stability [20]. The FES program consisted of three phases: warm-up, work phase, and recovery. Characteristics of the program were obtained from the user manual [21]. The pulse frequency was set to 3 Hz for warm-up and recovery phases. Both warm-up and recovery lasted for 5 min throughout the six-week program. During the work phase, the frequency alternated according to the following scheme: one cycle includes pulses of 8 Hz for 18 secs, then pulses of 2 Hz for 2 secs, and finally pulses of 18 Hz for 10 secs, with a pulse duration of 275 µs. The work phase lasted for 30 min for the first three weeks and for 40 min for the last three weeks. The intensity of the current was individually increased to the level at which strong visible muscle contraction was obtained, but with no unpleasant sensation reported by the participants. Muscles were activated simultaneously to generate co-activation, and therefore, to stiffen the trunk. To maintain correct alignment of the upper body during stimulation, the intensity of the current for one muscle was bilaterally equal.

### 2.3. Outcome Measures

#### 2.3.1. Muscle Tone

**Figure 1:**
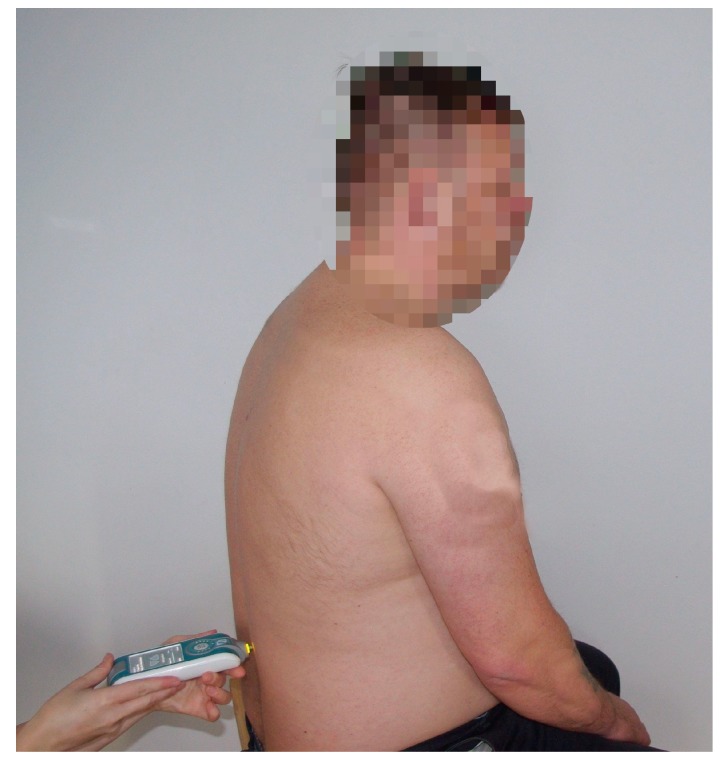
Measurement of erector spinae muscle oscillation frequency at L2 disc level using a MyotonPRO device.

The natural oscillation frequencies (MOF) for ES and RA muscles were measured in a sitting position on a custom-made adjustable chair, so that the participant had 90° of flexion in hip, knee, and ankle joints. Participants were asked to sit in a relaxed position, so that the measurements were taken with the muscles in a relaxed state. Measurement was carried out using a MyotonPro device (Myoton AS, Tallinn, Estonia) (Figure 1). Measurement points were marked on skin using a permanent marker by an experienced physical therapist, according to the MyotonPro user manual [22]. Measurement points of muscle belly for ES were marked at the L2 vertebrae level and for RA 2 cm bilaterally below the umbilicus. Measurements were performed prior to the first and last therapy session. Pre-pressure of 0.18 N was applied to a muscle, followed by a short duration (15 ms) mechanical impulse with a quick release of 0.40 N. The mechanical force applied to a muscle was 0.58 N (pre-load + impulse), hence, the minimal force did not cause residual mechanical deformation nor neurological reaction [22]. Each parameter was measured five times and the mean value was calculated and accepted for statistical analysis. If the coefficient of variation was more than 4%, the measurement was repeated.

#### 2.3.2. Dynamic Sitting Balance

Limits of stability (LOS; cm) were used to assess participants’ dynamic sitting balance. LOS can be defined as the maximum distance that a participant is able to move their COP in various directions while remaining stable and without changing the configuration of the base of support (BOS) [23]. Multidirectional LOS has been used to describe dynamic sitting balance in individuals with SCI [24].

Sitting balance parameters were measured with a CONFORMat sensor model 5530 (Tekscan, Inc., South Boston, USA) body pressure measurement system. The system was calibrated according to the manufacturer’s guideline prior to the testing, and then mounted on an adjustable chair. The chair height was individually adjusted considering the height of the participant. After sitting on the chair, the participants were instructed to keep their sitting area in contact with the chair and then to consecutively tilt their torso forward as far as possible in flexion, and then to the right (R) and left (L) in lateral flexion. Each motion cycle in the three directions was separated with a three second stabilization break. Three attempts were performed in each direction and mean data were accepted for statistical analysis. During the motions, the COP coordinates were acquired using the CONFORMat Research 7.20 software (Tekscan, Inc., South Boston, MA, USA) with a sample rate of 100 Hz. The COP data was then processed with Matlab (MathWorks, Inc., Natick, MA, USA) script to register the range of COP anterior–posterior and medio–lateral displacement in centimeters. In the current study, all analyzed dynamic sitting balance measures (cm) were normalized to participants’ body height (cm), and measured supine from cranium to calcaneus (LOS/body height).

MOF and LOS data from the last therapy session of the first intervention period were compared with the data obtained during the first therapy session of the second intervention period after a seven month break to see whether there had been a decline in the values.

### 2.4. Statistical Analysis

Data analysis was performed using IBM SPSS software (version 20, IBM Corporation, New York, NY, USA). Descriptive statistics were used to explain the demographics and SCI characteristics. As the sample size was very small, nonparametric tests were used for analyses. The Kruskal–Wallis H test was used to compare data between groups. Dunn´s post-hoc tests with Bonferroni correction were carried out for pairwise correction after a significant Kruskal–Wallis H test. As data from control group were collected for reference values, the baseline measures were also used for comparison of data with the SCI groups after the intervention. The Wilcoxon sign rank test was used for pre- and post-intervention comparison within one study group. The Wilcoxon test was also used to compare the outcome measures after the seven month break period. For the comparison after the seven month break period, all SCI participants formed one group (*n* = 5). Spearman’s rank correlation coefficient was calculated to assess the relationship between muscle tone and sitting balance. The relationship was calculated only at baseline. Data from the SCI participants’ (*n* = 5) first appointment were used for the analyses. The following correlation coefficient ranges were considered: 0.0–0.3 was considered negligible, 0.3–0.5 low; 0.5–0.7 moderate; 0.7–0.9 high; and 0.9–1.0 very high correlation [25]. A probability value (*p*-value) less than 0.05 was considered to be statistically significant. Hedges’s g value with 95% confidence intervals (CI) was calculated to describe the effect size of the intervention. A value of 0.2 was considered small, 0.5 medium, and over 0.8 was considered a large and substantial difference [26].

## 3. Results

**Figure 2:**
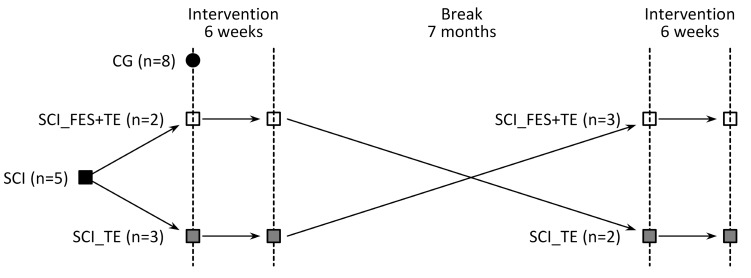
Schematic representation of crossover study design.

As crossover study design was used, where data from 5 SCI participants were included for analysis for both intervention groups. The representation of the study design is presented in Figure 2.

### 3.1. Muscle Tone

Results for muscle tone are presented in Table 2. Mean values for left and right body side were calculated and used for analysis.

After six weeks of intervention, MOF of ES increased 6.0% in SCI_FES+TE (*p* = 0.138), but decreased by 1.1% in SCI_TE (*p* = 0.500). Effect size values show that the therapy had a substantial effect on the SCI_FES+TE group. There was no significant difference of ES muscle tone between the three groups pre- (*p* = 0.989) and post-intervention (*p* = 0.553).

After six weeks of intervention, MOF of RA increased 6.1% in SCI_FES+TE group (*p* = 0.225) and 2.7% in SCI_TE group (*p* = 0.345). The therapy had a small effect on RA muscle tone in the SCI_FES+TE group. There was no significant difference of RA muscle tone between the three groups pre- (*p* = 0.542) and post-intervention (*p* = 0.329).

Within group comparison revealed that prior to the intervention, the MOF of ES was significantly higher than the MOF of RA in each of the three groups (SCI_FES+TE and SCI_TE *p* = 0.043; CG *p* = 0.018). After the intervention, a significant difference remained between MOF of ES and RA (SCI_FES+TE and SCI_TE *p* = 0.043; CG *p* = 0.018).

After the seven month break period, the MOF of ES decreased by 0.8% (*p* = 0.686, g = 0.16, 95% CI −1.08, 1.4). MOF or RA decreased by 1.4% (*p* = 1.000, g = 0.08, 95% CI −1.16, 1.32) in SCI participants.

### 3.2. Dynamic Sitting Balance

Results for dynamic sitting balance are presented in Table 2.

After six weeks of intervention, LOS of flexion increased 31.3% in SCI_FES+TE (*p* = 0.465), but decreased by 12.1% in SCI_TE (*p* = 0.345). Pre-intervention LOS of flexion was significantly lower for SCI_FES+TE (*p* = 0.022) as compared to CG. There was no statistical difference between SCI_TE and CG values pre-intervention (*p* = 0.056). Post-intervention LOS of flexion was not statistically different between SCI_FES+TE and CG (*p* = 0.059), but values for SCI_TE were significantly lower (*p* = 0.049) as compared to CG.

After six weeks of intervention, LOS of lateral flexion R increased 5.0% in SCI_FES+TE (*p* = 0.686) and 2.7% in SCI_TE (*p* = 0.465). Pre-intervention LOS of lateral flexion R was significantly lower (*p* = 0.049) for SCI_FES+TE as compared to CG. There was no statistically significant difference between the three study groups post-intervention (*p* = 0.054).

After six weeks of intervention, LOS of lateral flexion L increased 20.1% in SCI_FES+TE (*p* = 0.686) and 21.3% in SCI_TE (*p* = 0.500). Therapy had a small effect on both study groups. Prior to intervention, LOS of lateral flexion L was significantly lower for SCI_FES+TE (*p* = 0.042) and SCI_TE (*p* = 0.020) as compared to CG. After the intervention, no significant differences appeared between groups (*p* = 0.116).

After the seven month break period, LOS of flexion decreased by 31.9% (p = 0.138, g = 0.34, 95% CI -0.9, 1.59), LOS of lateral flexion R decreased by 27.3% (*p* = 0.225, g = 0.4, 95% CI −0.86, 1.65), and LOS of lateral flexion L decreased by 46.4% (*p* = 0.043, g = 0.99, 95% CI −0.33, 2.30).

### 3.3. Relationship between Muscle Tone and Dynamic Sitting Balance

A high negative correlation was found between MOF of ES and LOS of lateral flexion L. A moderate negative correlation was found between MOF of ES and LOS of flexion together with lateral flexion to R, and also between MOF of RA and LOS of flexion and lateral flexion R in SCI group (*p* > 0.05). Other analyzed correlation coefficients showed negligible to low correlation between muscle tone and dynamic sitting balance. Correlation values are presented in Table 3.

## 4. Discussion

Important findings of the study were as follows: combined therapy of FES and TE had a greater effect on increasing SCI participants’ muscle tone and improving their dynamic sitting balance than TE only; higher antagonist muscle tone had a negative impact on COP displacement characterizing dynamic sitting balance; after the seven month non-therapy period, trunk muscle tone and sitting balance characteristics decreased.

Spasticity is defined as “a motor disorder characterized by a velocity-dependent increase in tonic stretch reflexes (muscle tone) with exaggerated tendon jerks” [27]. Trunk muscle tone has to be high enough to resist gravity, but should be low enough to allow movement [28]. In the case of SCI, spasticity could also affect truncal muscles, causing abnormal posture [29]. In the current study, comparison between SCI participants and CG trunk muscle tone in a resting position showed that SCI participants’ MOF of ES and RA muscles was only slightly higher than in controls. There may have been more differences in tone characteristics if the measurements had been made during trunk muscles contractions, as the spasticity is velocity dependent, meaning the increase in muscle tone could only occur during movements.

One of the rehabilitation goals for SCI patients is to decrease spasticity, but it should be done within a limit, whereby the positive effect of spasticity still occurs [29]. Otherwise, it could be more difficult for SCI participants to sit unsupported and to perform daily activities. FES is usually used to decrease spastic muscle tone in spinal cord lesions [30], but the current study showed that resting tone does not differ between groups. A decrease in muscle tone was noted only in ES in the SCI_TE group, but the muscle tone characteristics were not significantly different between groups. It has been found that decrease in muscle tone while using FES occurs only when the muscle tone is initially high enough [31]. As the muscle tone was already low for the subjects, no further decrease was obtained. In the current study, increase in muscle tone was greater when FES was used. Although the increase was not statistically significant, the effect size values showed that the therapy had a substantial effect on ES muscle tone in the SCI_FES+TE group. This phenomenon may have occurred because therapeutic exercise and FES can also increase muscle tone. Similar results have also been obtained in previous research [32]. In a previous study, SCI participants’ thigh muscles were stimulated and the results showed an increase in resting muscle tone; therefore, the results are consistent with the current study. The increase in muscle tone has to be monitored during therapy, as the results of current study revealed that higher muscle tone has a negative impact on dynamic sitting balance. Therefore, if antagonist muscle tone is too high, the COP distance is reduced. Muscle tone has to be kept in an optimal state, whereby it does not interfere with movement but still helps to maintain posture against gravity.

Tonic and phasic muscle activity is needed for postural control, but as SCI participants’ muscles are partially or completely de-innervated [4] and the sensory information transmitted to the brain is decreased [33], their sitting balance is also decreased. Using FES in combination with TE showed better results in improving LOS of flexion. Changes were not significantly different, and although the percentage change (31.29%) was quite extensive, the effect size values revealed that the therapy had a small effect on LOS of flexion. Initially, there was a statistically significant difference between SCI_FES+TE and CG, but the increase in LOS of flexion was so extensive that the significant difference disappeared. LOS of R and L lateral flexion increased similarly in the two study groups. Although there was a slight increase in both study groups, effect size values demonstrated that there was almost no effect of the therapy on either group. However, the increase in SCI_FES+TE was still enough that the significant difference between SCI and CG groups disappeared. The reason why the results were not significantly better for the lateral flexion while using FES could be that FES stiffens the body, but during the exercises participants were supposed to move their trunk in different directions. In some cases, participants stated that stimulation was an interference and that it turned on at the wrong time; the participant wanted to move, but stimulation was keeping their trunk in place. Therefore, using stimulation that is not synchronized with exercises could be more beneficial for improving static sitting balance. The small increase in LOS of lateral flexion was probably because only ES and RA were stimulated. Activation of the quadratus lumborum muscle with stimulation has previously showed good results in mediolateral stability and side leaning positions [15]. Flexion and lateral flexion were used because most everyday activities are conducted in these directions. As participants were supported from behind while sitting in a wheelchair, the LOS of extension was not measured.

After the seven month break period, MOF of ES and RA decreased slightly, but the decrease in LOS characteristics in flexion and lateral flexion directions was extensive. However, a significant decrease was only noted in LOS of lateral flexion. The reason why MOF characteristics changed by only slightly over time could be that muscle tone is controlled by neural factors to a large extent [11,12]. Therefore, in the case of a central nervous system lesion, it is difficult to alter muscle tone over a long period of time with only exercises. Regular therapy could have an effect on muscle tone via intrinsic factors that the tone also depends on [11,12], but only during the time that therapy is conducted; the results do not increase over time. Participants’ dynamic sitting balance can be improved with training. Even if the participant has a severe lesion, other muscles can compensate for the movement, and to some extent, participants’ functional abilities can be improved. Therefore, the training had a bigger effect on LOS in different directions. However, during the seven month break period, when participants did not use FES or conduct exercises and did not use these maximum LOS values during their everyday activities, the results decreased. This shows that in order to keep the results, or even improve them, SCI participants need to exercise regularly, as the decrease in functional abilities is extensive over time.

Correlation analyses showed that in the SCI group, there was a low to moderate negative relationship between LOS directions and muscle tone. This finding indicates that the higher the muscle tone is, the smaller the LOS values are. This provides information for physical therapists, showing that while improving participants’ dynamic sitting balance, attention has to be paid to antagonist muscle tone.

The main limitation of the current study was the small sample size. Due to strict demands on participants and the limited region of the study, it was difficult to include enough participants. As the participants were very heterogeneous and the sample size was small, more research needs to be conducted to generalize the results.

## 5. Conclusions

A six week therapy program including combined FES and TE increases trunk muscle tone and dynamic sitting balance in flexion more than TE alone. Higher antagonist muscle tone negatively affects dynamic sitting balance and COP trajectory distance in various directions. After a seven month break period, a slight decline in trunk muscles tone values and extensive decrease in sitting balance values was noticed.

## Figures and Tables

**Table 1:** Participants’ anthropometric data, duration, and classification of spinal cord injury.

Variable	SCI	CG
Participants (*n*)	5	8
**Age (years)**		
Mean ± SD	39.2 ± 7.1	30.3 ± 7.9
95% CI	30.4, 48.0	17.7, 42.8
**Weight (kg)**		
Mean ± SD	76.9 ± 26.6	90.5 ± 14.4
95% CI	46.3, 107.4	67.6, 113.4
**Height (cm)**		
Mean ± SD	183.0 ± 8.8	184.9 ± 5.3
95% CI	172.1, 193.9	180.5, 189.4
**BMI (kg∙m^−2^)**		
Mean ± SD	23.0 ± 7.7	24.3 ± 4.1
95% CI	13.4, 32.7	20.8, 27.7
**Time Post Injury (years)**		
Mean ± SD	10.8 ± 6.0	N/A
95% CI	3.4, 18.2	
**Level of the injury**	C5–C6	N/A
**AIS—B/C (*n*)**	4/1	N/A

Abbreviation: SCI—spinal cord injury; CG—control group; AIS—American Spinal Injury Association Impairment Scale; BMI—body mass index; CI—confidence intervals; N/A—not applicable. Data include mean and standard deviation (SD).

**Table 2:** Mean (SD) pre- and post-intervention results for muscle tone (muscle oscillation frequency) and dynamic sitting balance (normalized limits of stability) with effect size (95% confidence interval) data.

	SCI_FES+TE (*n* = 5)			SCI_TE (*n* = 5)			CG (*n* = 8)
	Pre-Intervention	Post-Intervention	Effect Size	Pre-Intervention	Post-Intervention	Effect Size	Reference Value
**Muscle Oscillation Frequency (Hz)**							
ES	17.10 (1.05)	18.13 (1.07)	−0.88, (−2.18, 0.42)	17.56 (0.97)	17.36 (1.06)	0.18, (−1.06. 1.4)	16.99 (3.36)
RA	12.14 (1.37)	12.88 (2.04)	−0.38, (−1.64, 0.87)	12.34 (1.44)	12.88 (2.04)	−0.17, (−1.41, 1.0)	11.44 (1.66)
**Limits of Stability (cm, Normalized to Body Height)**							
Flexion	0.018 (0.016)	0.023 (0.023)	−0.23, (−1.47, 1.02)	0.022 (0.016)	0.020 (0.013)	0.12, (−1.12, 1.36)	0.049 (0.010)
Lateral flexion R	0.022 (0.015)	0.023 (0.017)	−0.06, (−1.3, 1.18)	0.021 (0.013)	0.021 (0.012)	−0.00, (−1.24, 1.24)	0.039 (0.008)
Lateral flexion L	0.018 (0.014)	0.022 (0.016)	−0.24, (−1.48, 1.00)	0.020 (0.010)	0.025 (0.011)	−0.43, (−1.68, 0.82)	0.038 (0.007)

Abbreviations: SCI—spinal cord injury; CG—control group; FES—functional electrical stimulation; TE—therapeutic exercise; ES—m erector spinae; RA—rectus abdominis; R—right; L—left.

**Table 3:** Spearman’s rank correlation coefficient for muscle tone and dynamic sitting balance. SCI participants’ pre-intervention data were used in correlation analysis.

Muscle Oscillation Frequency		
Limits of Stability	ES	RA
**SCI Group**		
Flexion	−0.600	−0.500
Lateral flexion R	−0.600	−0.500
Lateral flexion L	−0.700	−0.300
**CG group**		
Flexion	−0.252	0.120
Lateral flexion R	−0.071	0.286
Lateral flexion L	−0.333	−0.190

Abbreviation: SCI—spinal cord injury; CG—control group; ES—erector spinae; RA—rectus abdominis; R—right; L—left.
